# Serum IL-12 Is Increased in Mexican Obese Subjects and Associated with Low-Grade Inflammation and Obesity-Related Parameters

**DOI:** 10.1155/2013/967067

**Published:** 2013-02-20

**Authors:** K. Suárez-Álvarez, L. Solís-Lozano, S. Leon-Cabrera, A. González-Chávez, G. Gómez-Hernández, M. S. Quiñones-Álvarez, A. E. Serralde-Zúñiga, J. Hernández-Ruiz, J. Ramírez-Velásquez, F. J. Galindo-González, J. C. Zavala-Castillo, M. A. De León-Nava, G. Robles-Díaz, G. Escobedo

**Affiliations:** ^1^Unidad de Medicina Experimental, Hospital General de México “Dr. Eduardo Liceaga”, México D. F. 06726, Mexico; ^2^Laboratorio de Hígado, Páncreas y Motilidad, Departamento de Medicina Experimental, Facultad de Medicina, Universidad Nacional Autónoma de México, Hospital General de México “Dr. Eduardo Liceaga”, México D. F. 06726, Mexico; ^3^Departamento de Biología de la Reproducción y Clínica de Desórdenes de Sueño, Universidad Autónoma Metropolitana-Iztapalapa, México D. F. 09340, Mexico; ^4^Servicio de Medicina Interna, Hospital General de México, México D. F. 06726, Mexico; ^5^Servicio de Banco de Sangre, Hospital General de México, México D. F. 06726, Mexico; ^6^Fundación Mexicana para la Salud, México D. F. 14610, Mexico; ^7^Servicio de Cirugía General, Hospital General de México “Dr. Eduardo Liceaga”, México, 06726 DF, Mexico; ^8^Unidad de Desarrollo Biomédico, Centro de Investigación Científica y de Educación Superior de Ensenada (CICESE), Ensenada 22860, Baja California, Mexico

## Abstract

Interleukin-(IL-) 12 has been recently suggested to participate during development of insulin resistance in obese mice. Nevertheless, serum IL-12 levels have not been accurately determined in overweight and obese humans. We thus studied serum concentrations of IL-12 in Mexican adult individuals, examining their relationship with low-grade inflammation and obesity-related parameters. A total of 147 healthy individuals, 43 normal weight, 61 overweight, and 43 obese subjects participated in the study. Circulating levels of IL-12, tumor necrosis factor-alpha (TNF-**α**), leptin, insulin, glucose, total cholesterol, and triglyceride were measured after overnight fasting in all of the study subjects. Waist circumference and body fat percentage were recorded for all the participants. Serum IL-12 was significantly higher in overweight and obese individuals than in normal weight controls. Besides being strongly related with body mass index (*r* = 0.5154), serum IL-12 exhibited a significant relationship with abdominal obesity (*r* = 0.4481), body fat percentage (*r* = 0.5625), serum glucose (*r* = 0.3158), triglyceride (*r* = 0.3714), and TNF-**α** (*r* = 0.4717). Thus, serum levels of IL-12 are increased in overweight and obese individuals and show a strong relationship with markers of low-grade inflammation and obesity in the Mexican adult population. Further research is needed to understand the role of IL-12 in developing obesity-associated alterations in humans.

## 1. Introduction

Obesity is a public health problem of global dimensions [1], with an alarming high prevalence among the Mexican population [2]. Particularly, abdominal obesity is known to directly augment the risk to develop type 2 diabetes (T2D), cardiovascular disease, and other metabolic syndrome-associated alterations [3]. A growing body of evidence recently linked the pathogenesis of those disorders with a systemic low-grade inflammatory state [4], characterized by an increase in the circulating levels of several proinflammatory mediators such as tumor necrosis factor-alpha (TNF-*α*), C-reactive protein (CRP), and leptin [5, 6]. TNF-*α* has been shown to increase with adiposity in mice and humans [7], promoting impaired insulin sensitivity in adipose tissue, liver, and muscle [8]. CRP exhibits high circulating levels in overweight and obese subjects, having a positive correlation with increased body mass index (BMI) and waist-to-hip ratio [9]. Leptin is known to regulate food intake and body weight [10], as well as exert immunostimulatory actions including macrophage and T cell activation, dendritic cell (DC) maturation, and releasing of interferon-gamma (IFN-*γ*), TNF-*α*, and interleukin-(IL-) 6 [11], Thus, circulating proinflammatory factors have received increasing attention since they seem to have a major role during development of obesity-related disorders such as nonalcoholic steatohepatitis, T2D, and arteriosclerotic vascular disease (AVD) [3, 12].

IL-12 is a heterodimeric class-I helical cytokine that is mainly produced by DC and macrophages and influences differentiation of T helper 1 (Th1) immune cells [13]. IL-12 has been recently suggested to participate during development of obesity-related insulin resistance in rodents, since it clearly increases in both the epididymal adipose tissue and adipose tissue-associated proinflammatory macrophages in high-fat-diet-fed mice [14]. Moreover, IL-12 production can be directly stimulated by resistin, a proinflammatory adipokine that shows an important elevation in obese mice and T2D patients [15]. Nevertheless, serum IL-12 levels have not been precisely determined in subjects with a high metabolic risk such as both overweight and obese individuals.

We therefore evaluated circulating levels of IL-12 in Mexican normal weight, overweight, and obese individuals, examining their correlation with obesity-related parameters (fasting glucose, fasting insulin, insulin resistance, total cholesterol, total triglyceride, BMI, waist circumference, and body fat percentage) and low-grade inflammation markers (serum TNF-*α* and leptin).

## 2. Materials and Methods

### 2.1. Subjects

A total of 147 healthy Mexican adult volunteers from the south-central region of Mexico were included in the study. All of the participants provided written informed consent, previously approved by an institutional review board of the General Hospital of Mexico, which guaranteed that the study was conducted in accordance with the principles described at the Helsinki Declaration. Subjects were excluded from the study if they had previous or recent diagnosis of diabetes mellitus, cardiovascular diseases, chronic hepatic or renal disease, blood pressure higher than 140/90 mm Hg, inflammatory or autoimmune disorders, acute or chronic infectious diseases, cancer, and endocrine disorders. We additionally excluded pregnant or lactating women, subjects under any kind of cardiometabolic medication including anti-inflammatory, antiaggregant, and antihypertensive drugs, and subjects without having an overnight fasting of 8–12 hours. All of the individuals enrolled into the study received full medical evaluation, including achievement of clinic history and physical examination by a physician.

### 2.2. Anthropometric Measurements

According to the World Health Organization criteria for BMI, all of the participants were divided into three groups: normal weight subjects as the control group (BMI 18.5–24.9 kg/m^2^), overweight individuals (BMI 25–29.9 kg/m^2^), and obese subjects (BMI ≥ 30 kg/m^2^), where BMI is the result of dividing weight by height squared (kg/m^2^). Waist circumference was obtained from each study subject, considering the midpoint between the lower rib margin and iliac crest, using a conventional tape in centimeters (cm). For women, abdominal obesity was considered when their waist measurements were 80 cm or higher, whereas for men, abdominal obesity was considered when their waist measurements were 90 cm or higher. Body fat percentage was individually recorded by means of using a body composition analyzer (TANITA, Body Composition Analyzer, Model TBF-300A, Tokyo, Japan).

### 2.3. Biochemical Measurements

Blood samples were individually taken after overnight fasting and collected into pyrogen-free tubes (Vacutainer, BD Diagnostics, NJ, USA) at room temperature. Collection tubes were then centrifuged at 1000 g/4°C for 30 min, and serum samples were obtained and stored at −80°C in numerous aliquots until use. Total cholesterol and triglyceride were individually measured in triplicate by an enzymatic assay according to manufacturer's instructions (Roche Diagnostics, Mannheim, Germany). Serum insulin levels were individually determined in triplicate by means of the enzyme-linked immunosorbent assay (ELISA), following the manufacturer's instructions (Abnova Corporation, Taiwan). Serum glucose levels were individually determined in triplicate by the glucose oxidase assay, following the manufacturer's instructions (Megazyme International, Ireland). All of the biochemical measurements were performed at the same time in order to avoid procedural variations. The estimate of insulin resistance was individually determined by means of the HOMA-IR as follows: fasting insulin concentration (mU/L) × fasting glucose concentration (mmol/L) divided by 22.5.

### 2.4. IL-12, TNF-*α*, and Leptin Measurements

Blood samples were individually taken after overnight fasting and collected into pyrogen-free tubes (Vacutainer, BD Diagnostics, NJ, USA) at room temperature. Collection tubes were then centrifuged at 1000 g/4°C for 30 min, and serum samples were obtained and stored at −80°C in numerous aliquots until use. Serum levels of IL-12, TNF-*α*, and leptin were determined in triplicate by ELISA, following the manufacturer's instructions (Peprotech, Mexico). All of the cytokine measurements were performed at the same time in order to avoid procedural variations.

### 2.5. Statistical Analysis

Data from BMI, waist circumference, body fat percentage, fasting glucose, fasting insulin, HOMA-IR, total cholesterol, triglycerides, serum TNF-*α*, and leptin are expressed as mean ± standard deviation, using one-way ANOVA followed by a post hoc Tukey test for determining significant differences. Data from IL-12 are expressed as median and interquartile range in a box plot analysis, using the Kruskal-Wallis test or the Mann-Whitney *U* test for determining significant differences. The Spearman's correlation coefficient was performed for evaluating the relationship of IL-12 with anthropometric, biochemical, and inflammatory parameters. All of the studied groups were matched by gender and age. Statistical analysis was performed using the GraphPad Prism 5 software. Differences were considered significant when *P* < 0.05.

## 3. Results

A total of 147 participants were included into the study (43 normal weight controls, 61 overweight individuals, and 43 obese subjects). No significant differences were observed in age (for normal weight controls, mean age 30.3 ± 10.3 years; for the overweight group, mean age 32.4 ± 10.2 years; for the obesity group, mean age 34.7 ± 10.8 years) and woman and man proportion (26 women and 17 men in the normal weight control group; 30 women and 31 men in the overweight group; 21 women and 22 men in the obesity group) among all of the studied groups (Table 1). On the contrary, waist circumference, body fat percentage, fasting blood glucose, triglyceride concentration, and serum TNF-*α* showed a clear increase in overweight and obese individuals as compared with normal weight controls (Table 1). Total cholesterol, HOMA-IR, and leptin levels were significantly elevated in obese subjects as compared with normal weight individuals, but not with overweight subjects (Table 1). Furthermore, in our study population, BMI was strongly associated with waist perimeter (*r* = 0.896, *P* < 0.0001) and percentage of body fat (*r* = 0.700, *P* < 0.0001), while the latter ones also exhibited a positive significant relationship (*r* = 0.613, *P* < 0.0001).

**Figure 1:**
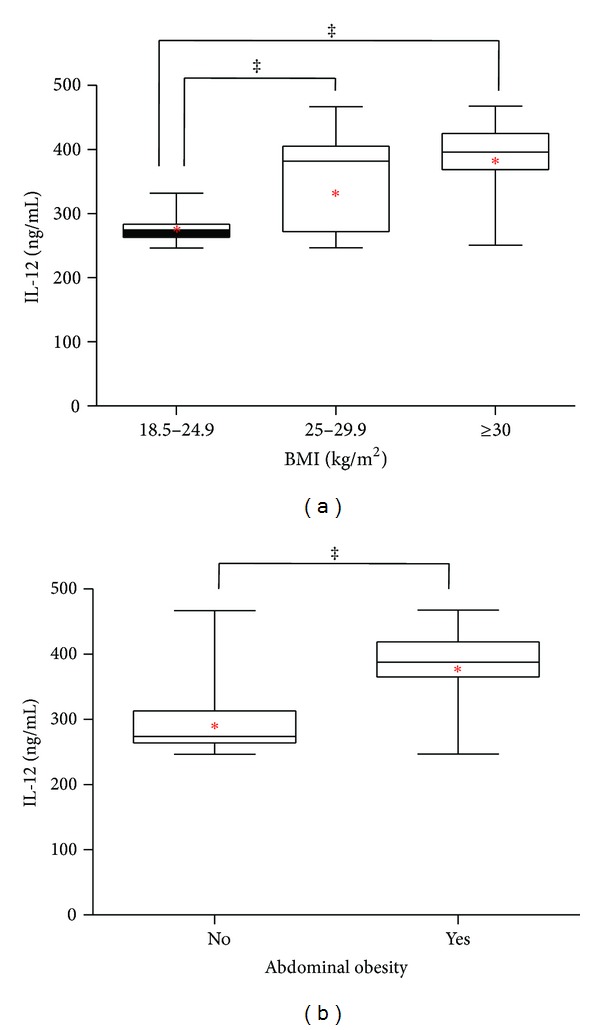
Serum levels of IL-12 in obese and nonobese individuals. Circulating levels of IL-12 (a) were assessed in normal weight, overweight, and obese subjects, according to the World Health Organization criteria for body mass index (BMI). In the study population, serum concentrations of IL-12 (b) were also evaluated in terms of abdominal obesity. Serum IL-12 was elevated in overweight and obese individuals, as well as in subjects exhibiting abdominal obesity. For women, abdominal obesity was considered when the waist measurement was 88 cm or higher, whereas for men, it was considered when the waist measurement was 102 cm or higher. Data are expressed as median and interquartile range in a box plot analysis. The mean value of IL-12 is graphically showed in a red asterisk. Differences were considered significant when *P* < 0.05.  ^‡^ *P* < 0.0001.

 In terms of BMI, serum levels of IL-12 exhibited a significant increase in overweight and obese subjects as compared to normal weight controls (Figure 1(a)). The mean value of IL-12 in overweight and obese individuals was 354.93 ± 68.39 and 382.04 ± 58.52, respectively, whereas in the normal weight group, it was 273.94 ± 16.05 (data expressed as mean ± standard deviation) (Figure 1(a)). Interestingly, serum IL-12 had a clear tendency to be significantly elevated in subjects exhibiting abdominal obesity, when compared with individuals showing a healthy waist circumference (Figure 1(b)). In this form, the mean value of IL-12 in subjects with abdominal obesity was 378.60 ± 66.86, while it decreases to 301.62 ± 59.81 in individuals with normal waist perimeter (data expressed as mean ± standard deviation) (Figure 1(b)).

**Figure 2:**
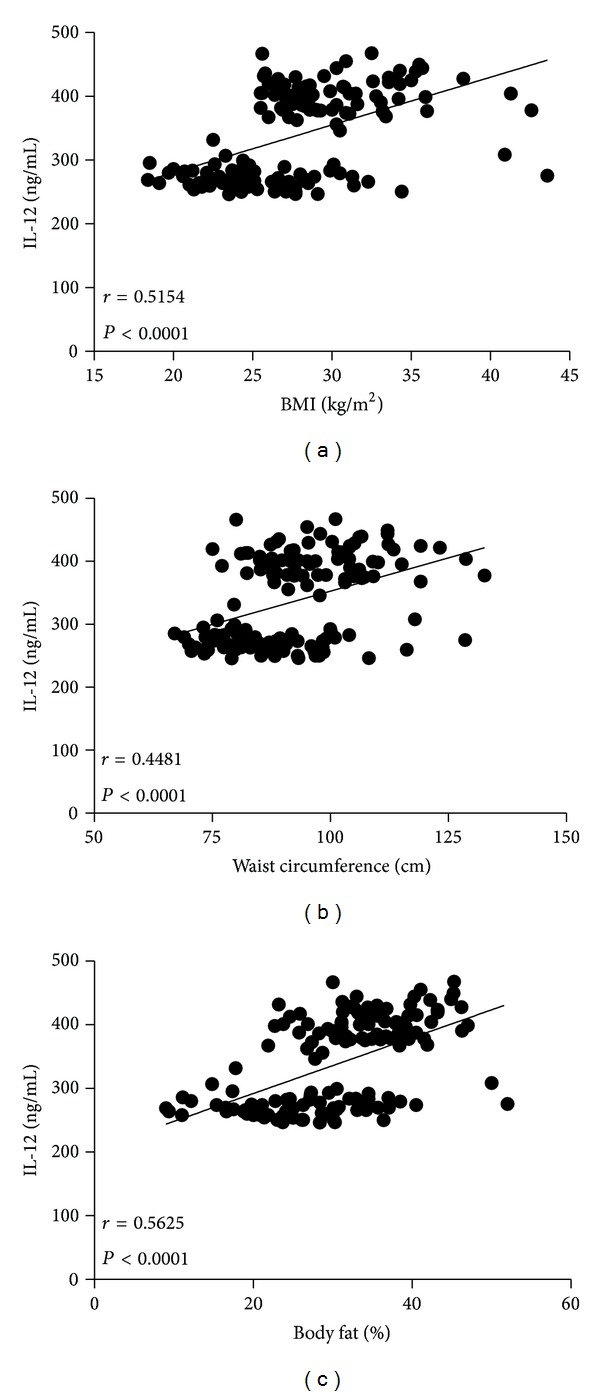
Relationship between serum levels of IL-12 and anthropometric parameters of obesity. Serum IL-12 values were positively associated with body mass index (BMI) (a), waist circumference (b), and body fat percentage (c). Coefficients (*r*) and *P* values were calculated by the Spearman's correlation model. The correlation level was considered significant when *P* < 0.05.

**Figure 3:**
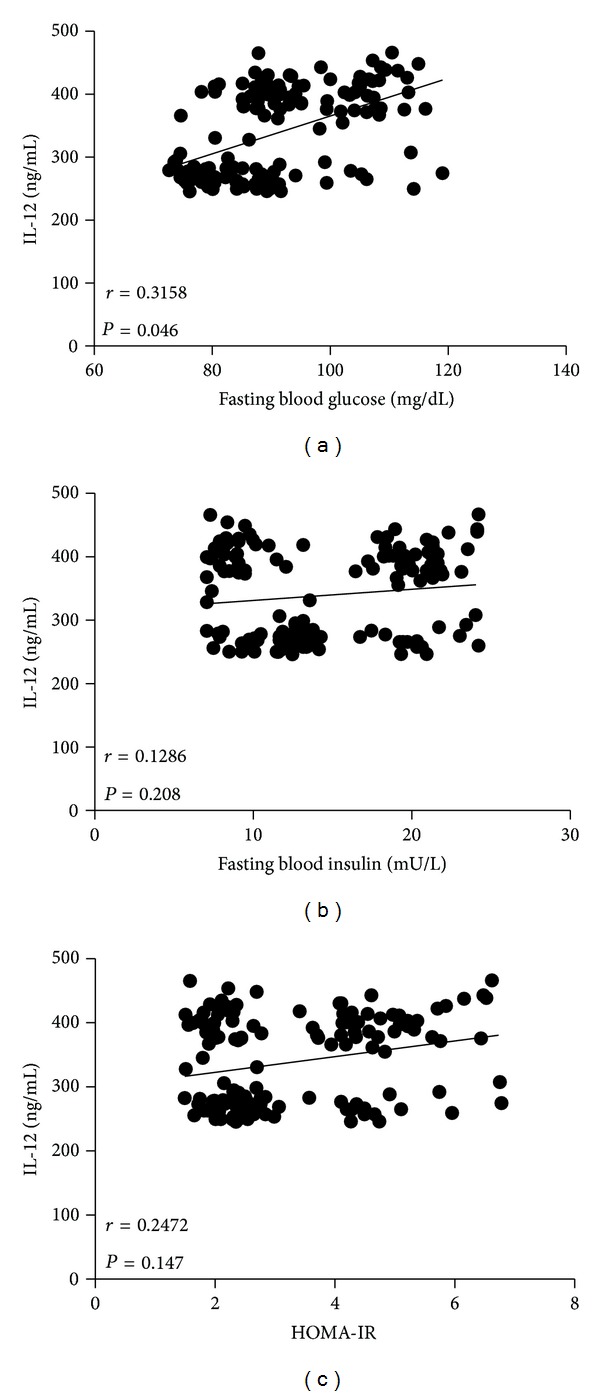
Relationship of the serum levels of IL-12 with fasting blood glucose, fasting blood insulin, and HOMA-IR. Serum IL-12 showed a positive significant association with high glucose levels (a), but not with insulin (b) or insulin resistance (c). The level of insulin resistance was estimated using the HOMA-IR index, which results from the next equation: fasting insulin concentration (mU/L) × fasting glucose concentration (mmol/L) divided by 22.5. Coefficients (*r*) and *P* values were calculated by the Spearman's correlation model. The correlation level was considered significant when *P* < 0.05.

**Figure 4:**
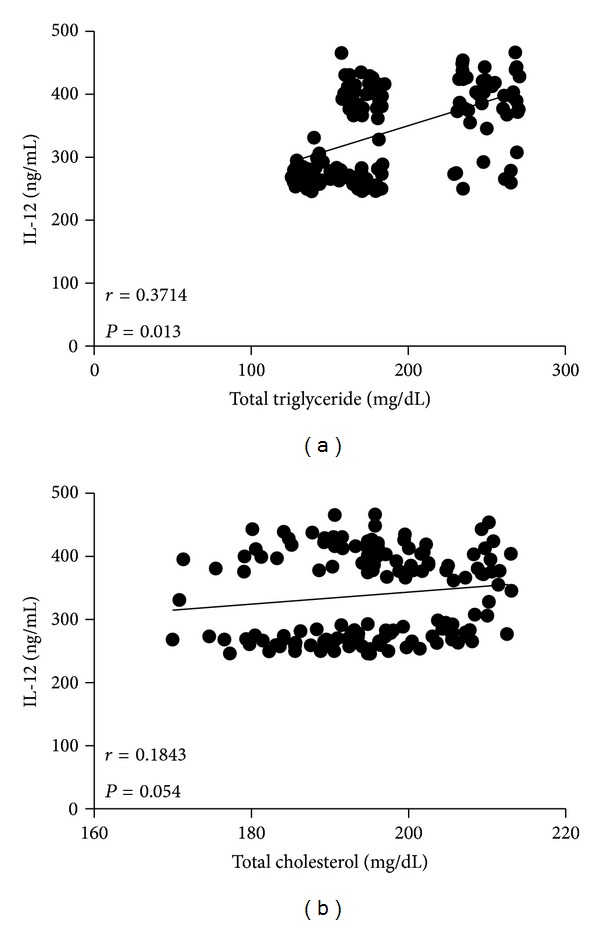
Relationship of the serum levels of IL-12 with total triglyceride and cholesterol levels. Serum IL-12 showed a positive significant association with high triglyceride levels (a), but not with total cholesterol (b). Coefficients (*r*) and *P* values were calculated by the Spearman's correlation model. The correlation level was considered significant when *P* < 0.05.

It was clear that while BMI and waist circumference augmented, circulating levels of IL-12 also increased in the study population. This result was denoted by a strong positive correlation between serum IL-12 and BMI (*r* = 0.5154, *P* < 0.0001) (Figure 2(a)), as well as IL-12 and waist circumference (*r* = 0.4481, *P* < 0.0001) (Figure 2(b)). Serum values of IL-12 also exhibited a positive relationship concerning percentage of body fat (*r* = 0.5625, *P* < 0.0001) (Figure 2(c)). Furthermore, circulating concentrations of IL-12 showed a clear association with other obesity-related parameters, including high serum glucose (*r* = 0.3158, *P* = 0.046) (Figure 3(a)) and increased triglyceride levels (*r* = 0.3714, *P* = 0.013) (Figure 4(a)). In contrast, no significant associations were observed among serum levels of IL-12 and fasting blood insulin (*r* = 0.1286, *P* = 0.208), insulin resistance denoted as HOMA-IR (*r* = 0.2472, *P* = 0.147), and total circulating cholesterol (*r* = 0.1843, *P* = 0.054) (Figures 3(b), 3(c), and 4(b), resp.).

**Figure 5:**
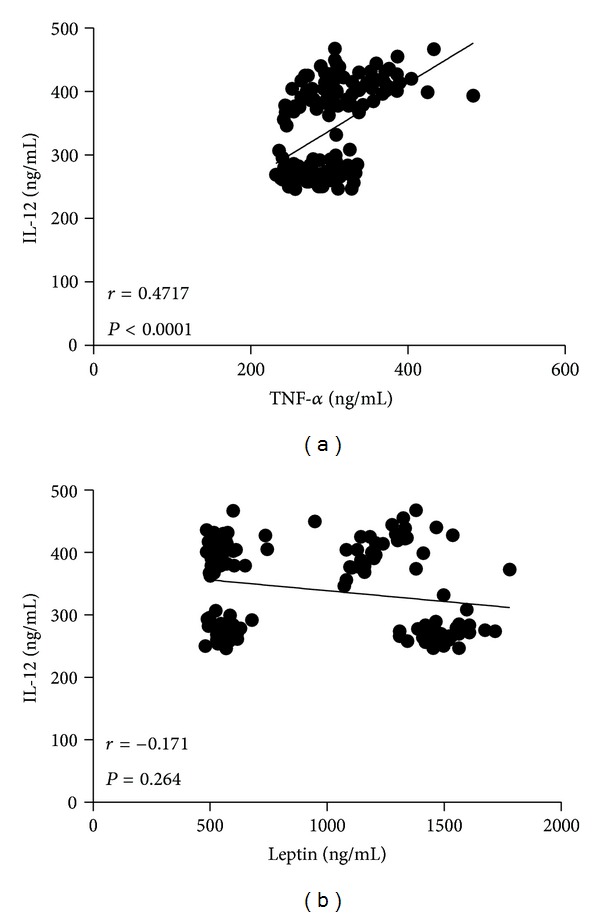
Relationship of the serum levels of IL-12 with systemic low-grade inflammation markers. Serum IL-12 showed a positive significant association with circulating levels of TNF-*α* (a), but not with leptin (b). Coefficients (*r*) and *P* values were calculated by the Spearman's correlation model. The correlation level was considered significant when *P* < 0.05.

In a similar way that in the case of anthropometric and biochemical parameters of obesity, serum IL-12 showed a significant coefficient of association with circulating levels of TNF-*α*, a typical inflammatory marker (*r* = 0.4717, *P* < 0.0001) (Figure 5(a)). On the contrary, despite the fact that leptin significantly increased in obese individuals as compared with control and overweight subjects (Table 1), no significant relationship was observed between this proinflammatory adipokine and IL-12 (*r = *−0.171, *P* = 0.264) (Figure 5(b)).

## 4. Discussion

IL-12 is one of the most representative members of the Th1 cytokine family, with well-known functions in inducing production of IFN-*γ* and differentiation of type 1 T cells, promoting in this way a linkage between innate response and adaptive immunity [13]. Nevertheless, recent experimental evidence from high-fat-diet-fed mice suggests that IL-12 could have an additional role in the systemic low-grade inflammation and the concomitant advent of obesity-related disorders, such as insulin resistance [14, 15]. For this reason, it is of much relevance to study the systemic levels of IL-12 in humans that show high metabolic risk, such as obese individuals. In this sense, it has been previously reported that circulating concentrations of IL-12 are significantly increased in subjects with metabolic syndrome [16] and type 2 diabetic patients undergoing cardiovascular complications [17]. Furthermore, peripheral blood mononuclear cells (PBMCs) from T2D patients are able to produce higher levels of IL-12 in response to lipopolysaccharide stimulation than those cells from healthy subjects [18]. In a similar sense, increasing IL-12 secretion has been also observed in human macrophages treated with resistin, a proinflammatory adipokine clearly elevated in obese individuals and T2D patients [15]. Interestingly, our data demonstrate that circulating levels of IL-12 start to increase in the overweight and have a strong relationship with central obesity, one of the key factors to develop obesity-related disorders such as metabolic syndrome and type 2 diabetes [1, 3]. Concomitantly, a recent study in a rural population of Mexican women suggests that the risk of having elevated serum IL-12 diminishes in women with high plasma concentrations of zinc, a micronutrient that has been related with a reduced risk for being obese [19]. In this sense, our results reveal that circulating concentrations of IL-12 increase at the same time that with parameters of obesity, including body mass index, body fat accumulation, and high glucose and triglyceride levels.

An interesting finding in this work is the relationship between serum levels of IL-12 and TNF-*α* in overweight and obese individuals. As a classical Th1 cytokine, IL-12 has the ability to induce IFN-*γ* production in differentiated T cells [13]. Interestingly, IFN-*γ* has been shown to increase in obese mice and humans [20, 21]. Thus, in view of the fact that it has been previously demonstrated that IFN-*γ* is able to promote releasing of TNF-*α* [4], it is plausible to expect that increasing levels of IL-12 correlate with high TNF-*α* levels in obese subjects. Our results seem to be consistent with this hypothesis, since serum IL-12 shows a strong positive relationship with circulating levels of TNF-*α* in both overweight and obese individuals. In this low-grade inflammation milieu, TNF-*α* has been extensively studied as a key inflammatory factor, capable to impair insulin signaling in murine adipocytes, through activation of negative regulators of the insulin receptor substrate 1-(IRS-1), including p38 mitogen-activated protein kinase and protein-tyrosine phosphatase 1B [22]. However, the serum levels of cytokines with the ability to induce production of TNF-*α* had not been precisely determined in obese humans to date. Therefore, as the low-grade inflammation seems to be determinant in the advent of obesity-related diseases such as type 2 diabetes and AVD [3, 4], further studies are needed in order to determine the role of IL-12 in the production of IFN-*γ* and TNF-*α* in obese subjects.

On the other side, unexpectedly, we did not observe a significant statistical correlation between serum IL-12 and leptin, despite the fact that leptin showed a clear increase in obese subjects. Nevertheless, it has been shown that leptin does not always correlate with other low-grade inflammation markers. For instance, a previous study conducted in Serb obese women reported a significant augmentation in the plasma levels of the proinflammatory cytokines IL-17 and IL-23, independently of the increase in leptin [23]. In a similar way, a Th1-immune profile characterized by an increase in the circulating proportion of IFN-*γ*-secreting PBMC has been described in Italian obese children, without a significant relationship with leptin levels [24]. Consistent with previous studies [19], present results suggest that leptin may not be significantly related with the systemic mild-grade inflammation milieu in this study population. However, additional research taking into consideration the influence of age-, sex-, and ethnicity-associated factors upon leptin levels is required in order to draw major conclusions. A similar result was observed in the absence of significant association of IL-12 with insulin, total cholesterol, and insulin resistance. Regarding this issue, it has been previously reported that the relationship of IL-12 with elevated insulin resistance and dyslipidemia increases in subjects with type 2 diabetes and AVD [17]. Since we have shown that IL-12 does not seem to have a significant relationship with insulin resistance and total cholesterol in healthy obese subjects, it is probable that IL-12 could be more related with the advent of obesity-related complications than with obesity itself. Nevertheless, further studies using a larger study population of healthy, prediabetic, and diabetic obese subjects are necessary for a more accurate determination of the relationship of IL-12 with insulin resistance and hypercholesterolemia in humans.

Finally, it is worth to mention that serum levels of IL-12 seem to be grouped in two clusters in our study population, characterized by high and low production of IL-12. Interestingly, there are no high producers of IL-12 among normal weight subjects. However, increased production of IL-12 seems to start since the overweight, reaching a plateau in the obesity group. It is important to underline that more than 70% of the high producers of IL-12 also showed increased levels of triglycerides (data not shown). Since there was no difference in the serum levels of IL-12 between women and men (data not shown), we hypothesize that high production of IL-12 may be a marker related to body weight gain and triglyceridemia. Nevertheless, taking into consideration that several factors are able to influence high and low production of cytokines [25–27], further research is required to conclude whether body weight gain and triglyceridemia are determinant factors in the production of IL-12 in humans.

## 5. Conclusions

In conclusion, the present study demonstrates that serum levels of IL-12 are significantly higher in overweight and obese individuals than in normal weight subjects. In the Mexican adult population, serum levels of IL-12 show a strong relationship with systemic low-grade inflammation and obesity-related markers, including TNF-*α*, abdominal obesity, percentage of body fat, and high glucose and triglyceride levels. Additional research should be addressed in order to understand the role of Th1 cytokines in developing of insulin resistance, dyslipidemias, cardiovascular disease, and other obesity-associated alterations in humans.

## Figures and Tables

**Table 1:** Anthropometric and biochemical characteristics of the study subjects.

	BMI			*P* value
	18.5–24.9^a^	25–29.9^b^	≥30^c^
Gender (W/M)	26/17	30/31	21/22	N. S.
Age (years)	30.3 ± 10.3	32.4 ± 10.2	34.7 ± 10.8	N. S.
BMI (kg/m^2^)	22.7 ± 1.8	27.4 ± 1.2	33.6 ± 3.4	^ b,c^ *P* < 0.0001
Waist circumference (cm)	80.1 ± 6.9	91.2 ± 6.8	107.2 ± 9.7	^ b,c^ *P* < 0.0001
Body fat percentage	24.2 ± 8.1	30.3 ± 6.1	37.5 ± 6.9	^ b^ *P* < 0.05, ^c^ *P* < 0.0001
Fasting blood glucose (mg/dL)	78.9 ± 5.2	88.7 ± 3.6	106.1 ± 7.4	^ b^ *P* < 0.05, ^c^ *P* < 0.0001
Fasting blood insulin (mU/L)	12.23 ± 2.65	15.30 ± 7.94	16.84 ± 8.20	N. S.
HOMA-IR	2.58 ± 0.51	3.24 ± 0.36	3.81 ± 0.59	^ c^ *P* < 0.05
Total cholesterol (mg/dL)	187.2 ± 7.6	195.8 ± 6.9	203.5 ± 8.1	^ c^ *P* < 0.05
Total triglyceride (mg/dL)	136.5 ± 9.4	172.3 ± 11.8	251.3 ± 19.5	^ b,c^ *P* < 0.0001
TNF-*α* (ng/mL)	256 ± 16.6	289.7 ± 37.5	341.2 ± 45.5	^ b^ *P* < 0.05, ^c^ *P* < 0.0001
Leptin (ng/mL)	558.1 ± 43.5	545.5 ± 58.8	1246.3 ± 149.6	^ c^ *P* < 0.0001

W: women; M: men; BMI: body mass index; HOMA-IR: homeostatic model assessment-insulin resistance; TNF-*α*: tumor necrosis factor-alpha.

Data are presented as mean ± standard deviation. Differences were considered significant when *P* < 0.05, as comparing overweight (b) and obesity (c) groups with normal weight subjects (a).
